# Associations of Serum Cartilage Oligomeric Matrix Protein with Body Composition and Hematological Inflammatory Indices in Postmenopausal Women

**DOI:** 10.3390/ijms27198831

**Published:** 2026-10-03

**Authors:** Michał Wojciechowski, Alicja Nowak, Małgorzata Ogurkowska, Tomasz Podgórski, Piotr Leszczyński, Anna Huta-Osiecka, Krystian Wochna

**Affiliations:** 1 Department of Biochemistry, Poznan University of Physical Education, 61-871 Poznan, Poland; 2 Department of Biomechanics, Poznan University of Physical Education, 61-871 Poznan, Poland; 3 Department and Clinic of Internal Medicine and Metabolic Disorders, Poznan University of Medical Sciences, 61-701 Poznan, Poland; 4 Greater Poland Center of Pulmonology and Thoracic Surgery, 60-569 Poznan, Poland; 5 Department of Swimming and Water Lifesaving, Poznan University of Physical Education, 61-871 Poznan, Poland

**Keywords:** cartilage oligomeric matrix protein (COMP), menopause, lean body mass, body fat, systemic inflammation

## Abstract

In the postmenopausal period, a decrease in sex hormone levels may alter body composition and can predispose individuals to increased body fat and musculoskeletal tissue degeneration, including a deterioration in the articular cartilage. The aim of the study was to assess the resting serum cartilage oligomeric matrix protein (COMP) concentrations in postmenopausal women with different body mass indexes (BMIs) and how COMP correlates with both lean and fat mass as well as with blood count-derived inflammatory markers. Thirty-three postmenopausal women were included in the study. Total fat and lean body mass were measured via dual-energy X-ray absorptiometry. Blood serum concentrations of COMP and adiponectin were assessed, and lymphocyte, monocyte and granulocyte counts were determined. The lymphocyte-to-monocyte ratio (LMR) and granulocyte-to-lymphocyte (GLR) ratios were calculated. The analysis of differences in body mass, BMI, lean body mass, fat mass, and android (*p* ≤ 0.01) and gynoid fat mass (*p* < 0.05) revealed significantly lower values in the group of women with a normal BMI compared to the group with a BMI above the normal range. In the group of women with a normal BMI, a significant relationship was noted between COMP and lean body mass (*p* ≤ 0.01). In the group of women with a BMI above the normal range, a significant positive correlation of COMP with LMR (*p* < 0.05) and a negative correlation with GLR (*p* < 0.05) were observed. The significant associations between resting serum COMP levels and lean body mass and inflammatory markers, which varied according to BMI categories, suggest that body composition may be an important factor to consider when using COMP for diagnostic purposes.

## 1. Introduction

Apart from age, excess fat mass can influence metabolic and immune system dysregulation and may lead to musculoskeletal tissue degeneration [1]. In the postmenopausal period, a decrease in sex hormone levels may contribute to changes in body composition, including increased adiposity and reductions in bone and skeletal muscle mass [2]. The results of numerous studies indicate a relationship between being obese and the prevalence of joint diseases. It was found that obesity plays a key role in the predisposition to osteoarthritis, both through mechanical load and metabolic and inflammatory processes [1,3]. Recent research emphasizes the important role of metabolic abnormalities and systemic low-grade inflammation in the pathogenesis of joint disorders [4].

Particular attention has been paid to biologically active adipokines such as leptin, visfatin, resistin and chemerin, as well as cytokines, including TNF-α and IL-6, produced by adipose tissue and involved in the pathogenesis and progression of degenerative musculoskeletal diseases [1,5]. Adipokines exhibit both pro- and anti-inflammatory properties and play a role in metabolic and inflammatory disturbances [6]. The elevated release of these molecules from adipose tissue in obese people is considered an important factor contributing to cartilage loss by stimulating the production of cytokines, matrix metalloproteinases (MMPs) and prostaglandins, as well as inhibiting the synthesis of proteoglycans and type II collagen. Adipokine secretion from adipose tissue is thought to link obesity and osteoarthritis [7]. Adiponectin, produced by white adipose tissue, has different physiological roles depending on its isoform and effector tissue, exhibiting both pro-inflammatory and anti-inflammatory effects [8]. However, the role of this molecule in the pathogenesis of joint degeneration is controversial [9].

Systemic inflammation may influence hematopoiesis in the bone marrow, leading to alterations in peripheral blood cell counts. Unfavorable changes in hematological parameters have been observed in people with excessive fat mass [10]. Recent studies highlight the role of complete blood count-derived inflammatory markers as indicators of immune dysregulation, such as the neutrophil-to-lymphocyte ratio (the most abundant type of granulocyte, GLR), or lymphocyte-to-monocyte ratio (LMR). Previous studies have confirmed that lymphocyte and neutrophil counts might be considered an acute inflammatory response to chronic inflammation [11]. Moreover, LMR has been a subject of great interest in a wide range of immunology studies [12], with some authors noting relationships between these indices and cartilage condition [13].

Cartilage oligomeric matrix protein (COMP) is a non-collagenous extracellular matrix glycoprotein whose primary role is to maintain the stability of the collagen network [14]. Serum COMP concentration serves as a diagnostic biomarker for identifying articular cartilage metabolism, and most study results indicate elevated COMP levels in patients diagnosed with osteoarthritis compared to the control group [15,16]. However, COMP is also expressed in several other tissues, and recent studies indicate its involvement in diseases, such as cancer, cardiovascular diseases, and tissue fibrosis [14]. This may be of particular interest given that circulating COMP concentrations have been positively associated with BMI and adiposity [17]. Nevertheless, the relationship between circulating COMP concentrations and body composition in healthy individuals at rest remains insufficiently understood. Therefore, the purpose of the study was to assess the resting serum COMP concentrations in postmenopausal women with different body mass indexes (BMI) and their associations with lean and fat mass and also with LMR, GLR and adiponectin status.

## 2. Results

Table 1 presents the basic somatic characteristics and biochemical indices for both groups of women: with a BMI above the normal range and with a normal BMI. A comparative analysis of these variables between the women’s groups revealed significantly lower values of body mass, BMI, lean body mass, fat mass, and android (*p* ≤ 0.01) and gynoid fat mass (*p* < 0.05) in the group with a normal BMI compared to the group with a BMI above the normal range.

For both groups of women classified based on BMI values, a correlation analysis was carried out to evaluate the relationships between the levels of COMP and other variables (Table 2). In the group of women with a normal BMI, the only significant correlation identified was between COMP and lean body mass (*p* ≤ 0.01). In the group of women with a BMI above the normal range, a significant positive correlation of COMP with LMR (*p* < 0.05) and a negative correlation with GLR (*p* < 0.05) and physical activity levels (hours/week; r = −0.72; *p* = 0.0455) were observed.

Variables that showed significant correlations with serum COMP concentrations were included in the regression model. In the group of women with normal BMI, a linear regression analysis revealed a statistically significant association between COMP and lean body mass. The regression model was statistically significant (F(1,23) = 8.36; *p* = 0.008), and the coefficient of determination was R^2^ = 0.27, indicating that 27% of the variance in COMP levels was explained by the model.

In the group of women with a BMI above the normal range, the linear regression analysis revealed statistically significant relationships between COMP and both LMR and GLR. In the case of LMR, the regression model was statistically significant (F(1,6) = 13.39; *p* = 0.0110), and the coefficient of determination was R^2^ = 0.69, indicating that 69% of the variance in COMP levels was explained by the model. In the case of GLR, the regression model was statistically significant (F(1,6) = 6.95; *p* = 0.0387), and the coefficient of determination was R^2^ = 0.54, indicating that 54% of the variance in COMP levels was explained by the model.

## 3. Discussion

The present study investigated the relationship between serum COMP concentration and body mass and body composition. Our findings revealed no significant correlations between this marker and either body mass or BMI. However, in studies by other authors, associations between these variables were observed [17,18]. Lambova et al. [18] assessed serum COMP concentrations at rest in patients with metabolic osteoarthritis of the knee and in a control group, with both patients and the controls having varying BMI values. Their results showed significantly lower levels of this marker in patients compared with the control group; however, COMP levels in the control group were significantly higher in overweight and obese individuals than in those with a normal BMI. Similar results in women and men were observed by Denton et al. [17] (BMI range: 18.8–46.2 kg/m^2^). It should be noted that, unlike those studies, in our study, we did not include any participants with severe obesity, and the highest BMI value was 33 kg/m^2^. Moreover, the classification of women into groups was based on their BMI, taking into consideration their age. It is possible that this is why we did not observe any significant variation in COMP concentration based on BMI values.

We observed a significant correlation between serum COMP concentrations and lean body mass in the group of women with a normal BMI. However, we did not find such a correlation in the group of women with BMI values above the normal range. One possible explanation for this finding is that excess adipose tissue acts as an endocrine organ, releasing pro-inflammatory mediators [5], which could modify COMP levels. Moreover, adipose tissue has been identified as a site of COMP expression [17]. In the study by Denton and colleagues [17], COMP mRNA levels in adipose tissue (abdominal and gluteal) and plasma COMP concentrations correlated positively with overall obesity. However, in our study, we did not observe any significant correlations between COMP concentration and fat mass in either of the studied groups.

The relationship between COMP concentrations and lean body mass in women with a normal BMI may be explained by two mechanisms. Firstly, the higher resting COMP levels may be associated with larger tissue volumes (e.g., muscles, bone and cartilage). Skeletal muscle, a major component of lean body mass, may contribute to the production of myokines, including irisin, which has been implicated in the regulation of chondrocyte and cartilage matrix metabolism, supporting the concept of muscle–cartilage cross-talk [19]. Secondly, increasing muscle strength gives the joints greater support, while strengthening muscles is one of the key ways to help protect joints [20]. In our study, we did not observe a significant difference in serum COMP levels between groups classified according to BMI values; however, it is worth noting that women with a BMI above the normal range not only had a higher percentage of body fat but also a higher lean body mass compared to the first group of women (with normal BMI values). In their cross-sectional study of postmenopausal women, Kim et al. [2] observed that sarcopenia is an important factor in knee osteoarthritis in obese subjects compared to obesity without sarcopenia. Consequently, they concluded that obesity may have a greater negative impact on cartilage only when it coexists with sarcopenia.

In our study, we assessed serum COMP concentration only at rest. In studies analyzing the effect of mechanical stimuli during physical activity on blood COMP levels in healthy subjects, an increase in this marker was most commonly observed [21,22]. The change in serum COMP concentrations was associated with the load magnitude, for example, running versus swimming. However, the origin of increased serum COMP levels after exercise remains unclear [23]. Erhart-Hledik et al. [24] showed that in patients with knee osteoarthritis, the magnitude of change in COMP concentration after mechanical stimulation (30 min walking activity) was associated with cartilage thinning over a 5-year follow-up. Therefore, they suggested that changes in COMP levels after mechanical stress may express the health status of cartilage. In the study by Herger et al. [22], conducted in a population of individuals after anterior cruciate ligament (ACL) injury (2 to 10 years after the injury) and in healthy individuals, mechanical stress in the form of treadmill walking caused a change in serum COMP concentration in both groups. However, the authors observed that the magnitude of this load-induced COMP increase correlated with cartilage MRI T2 relaxation times only in individuals with the ACL injury, and not in the healthy controls. This suggests that load-induced changes in COMP serum concentration kinetics may reflect an altered metabolic response to mechanical load in knee cartilage in patients after ACL injury.

Our study found a significant association between COMP and indices of immunological state, but only in the group of individuals with a BMI above the normal range. Although we did not measure cytokine levels, inflammation-related, blood count-derived inflammatory markers, such as LMR and GLR, were determined. We observed relationships between COMP and these indices in opposite directions. Given the small sample size, the regression estimates may be unstable and should therefore be interpreted with caution and considered hypothesis-generating rather than as definitive evidence of an association.

Previous studies have confirmed that neutrophil (the most abundant type of granulocyte) and lymphocyte counts might be considered an acute inflammatory response to chronic inflammation [11,12]. The neutrophil-to-lymphocyte ratio was identified as a marker of inflammatory state in obesity and positively correlated with waist circumference [11]. Low-grade inflammation plays a role in cartilage degeneration, and Stannus et al. [25] found that serum levels of IL-6 and TNF-α were associated with knee cartilage loss in older people. In obese subjects, Zhang et al. [13] observed that LMR was negatively associated with OA risk, while inflammatory indicators such as the neutrophil-to-lymphocyte ratio or C-reactive protein were associated with an increased risk of osteoarthritis. In addition, Wang et al. [7] suggested that LMR may be associated with a good axial spondyloarthritis (SpA) prognosis because a higher LMR could result in reduced effects of TNF-α. In our study within small subgroups, linear regression analysis showed that the serum COMP level is determined by LMR and GLR (69% and 54% of the variance, respectively). Therefore, we suppose that these factors could contribute to the lack of correlation between COMP and lean body mass in subjects with BMI above the normal range.

We also measured adiponectin levels, as this adipokine acts as an anti- and pro-inflammatory factor [8]. However, its role in the pathogenesis of OA remains controversial [1,5,9]. In a large-population cross-sectional study, serum adiponectin levels were positively correlated with the radiographic severity of knee osteoarthritis [9]. Our study, however, did not reveal any significant differences in the levels of this adipokine between the two groups of women, nor any correlation between serum adiponectin and COMP concentrations.

Although a negative relationship between resting serum COMP concentrations and the weekly duration of physical activity was observed in the group of women with a BMI above the normal range, this finding should be interpreted with caution given the small number of participants. Studies investigating physical activity’s influence on cartilage health have yielded mixed results. Petrigna et al. [26] investigated 24 studies regarding physical activity interventions and concluded that exercise programs predominantly had a positive impact on cartilage structure. However, they suggested that when prescribing such interventions, specialists should be careful to select the most appropriate exercises based on the patient’s needs.

Although physical activity is recommended for maintaining joint cartilage health, obese individuals may experience excessive stress and pain on their loaded joints during movement. Obesity’s cumulative effect on joint loads during daily activities is thought to play a critical role in disease progression [3]. Peishun et al. [27] found that power bicycle training can prevent a decrease in knee joint cartilage volume, while moving treadmill training can decrease it in patients with obesity. Therefore, given the complex effects of pro-inflammatory factors and mechanical stress on the condition of articular cartilage in people with an increased BMI, it can be speculated that caution should be taken when recommending forms of physical activity.

One limitation of the study is the relatively small sample size. Future studies should also assess musculoskeletal status using biomechanical methods. Moreover, we did not use direct measurements of daily physical activity. In addition, evaluating the nutritional status of study participants is essential, since adequate protein intake affects joint health. In an animal model, Yamada et al. [28] observed that chicken leg extract promoted cartilage matrix production. Moreover, nutrients are an important factor influencing lean body mass in older adults, and low protein intake is associated with sarcopenia. A daily protein intake of 1.0–1.2 g per kg of body weight, or 25–30 g of high-quality protein per meal, is recommended; the importance of adequate energy intake is also emphasized [29]. In older adults, nutritional behaviors associated with malnutrition correlate with less lean mass and greater fat mass and body fat percentage [30].

## 4. Materials and Methods

### 4.1. Participants

Thirty-three postmenopausal women, aged 58–75 and declaring no use of hormone replacement therapy, were included in the study. The survey and interview questionnaire provided information on lifestyle and diseases. Participants who declared diabetes mellitus or cancer, or who were undergoing hormone replacement therapy, were not included in the study. Eight women from the group with normal BMI values reported taking medication for osteoporosis or/and having degenerative changes in certain joints. However, these women were retained in the analysis, as the association between COMP concentration and lean body mass remained significant even after their exclusion (n = 25, r = 0.52, *p* = 0.0080 vs. n = 17, r = 0.54, *p* = 0.0253). Furthermore, a comparative analysis (*t*-test) revealed no significant difference in COMP concentration between the group of these women (n = 8) and the group of women who did not report osteoporosis or osteoarthritis (n = 17). All participants provided written consent to participate in the study. The research was approved by the Bioethics Committee at the Poznan University of Medical Sciences (code no. 245/19).

### 4.2. Body Composition Measurements

Total fat and lean body mass were determined using dual-energy X-ray absorptiometry (DXA). DXA measurements were acquired using a Lunar Prodigy Advance densitometer (General Electric, Boston, MA, USA).

### 4.3. Biochemical Analysis

For biochemical analysis, venous blood was drawn after fasting between 7:30 and 9:30 am. Blood was collected using two tubes. The S-Monovette tube with a clotting activator (Sarstedt, Nümbrecht, Germany) was centrifuged to obtain the serum and stored at −70 °C until biochemical analyses were performed. The enzyme-linked immunosorbent assay (ELISA) method was applied to determine concentrations of the human COMP and adiponectin (BioVendor, Brno, Czech Republic, sensitivity 0.4 ng/mL and 26 ng/mL, respectively) using a multi-mode microplate reader (Synergy 2 SIAFRT, BioTek, Winooski, VT, USA).

To determine lymphocytes, monocytes, and granulocytes, blood was collected using S-Monovette tubes (Sarstedt, Nümbrecht, Germany) containing K2-EDTA (EDTA dipotassium salt) as an anticoagulant. Hematological measurements were carried out using the 20-parameter automated hematology analyzer Mythic^®^ 18 (Orphée, Geneva, Switzerland). The lymphocyte-to-monocyte ratio (LMR) and granulocyte-to-lymphocyte ratio (GLR) were calculated.

### 4.4. Statistical Analysis

Statistical analyses were conducted for the entire group of women (n = 33). Following the identification of a significant association between COMP concentration and lean body mass, the participants were divided into two subgroups according to BMI categories, in accordance with the recommendations of the Committee on Nutrition and Health: the group with normal BMI values (n = 25; BMI 23–28 kg/m^2^ for women aged 55–65 years and BMI 24–29 kg/m^2^ for women aged >65 years) and the group with BMI above the normal range (n = 8) [31].

The normality of distribution was evaluated using the Shapiro–Wilk test, while the homogeneity of variances between the compared groups was assessed using Levene’s test. The differences in variables between the groups of women were established using the following statistical procedures: the independent Student *t*-test for variables meeting both the assumptions of normality and homogeneity of variance (*t*-test); Welch’s *t*-test (with separate variance estimates) for normally distributed variables that violated the assumption of equal variances; and the Mann–Whitney U test as a non-parametric alternative for variables that failed to meet the normality assumption. Pearson analysis for normally distributed variables and Spearman’s rank analysis for non-normally distributed variables were used to calculate correlation coefficients. *p*-values obtained from the correlation analyses were adjusted using the Benjamini–Hochberg procedure to control the false discovery rate (FDR) due to multiple testing. For normally distributed variables, simple linear regression models were applied to evaluate relationships between COMP and dependent variables. The influence of individual observations on the fitted regression models was assessed using Cook’s distance, with potentially influential observations identified based on this measure. Statistical significance was set at *p* < 0.05 for all analyses. All statistical procedures were conducted using Statistica software (version 13.3, TIBCO Software Inc., Tulsa, OK, USA).

## 5. Conclusions

The significant association between resting serum COMP concentrations and lean body mass, particularly in women with a normal BMI, may indicate that the condition of the musculoskeletal system plays a significant role in determining the level of this marker. Conversely, in women with a BMI above the normal range, immune status affects COMP level to a greater extent. These results suggest that body composition may need to be taken into account when using this marker for diagnostic purposes. We highlight the importance of further research in this area involving larger study populations and a broader range of age groups.

## Figures and Tables

**Table 1:** Somatic characteristics and biochemical indices for groups of women with normal BMI and with BMI above the normal range and a comparative analysis between these groups.

Parameters	Group with BMI Above Normal Range	Group with Normal BMI	*p*-Value
Age (years)	66.75 ± 4.06	66.40 ± 4.20	0.8377 ^a^
Age of menopause (years)	49.88 ± 4.94	50.32 ± 4.41	0.8108 ^a^
Physical activity (h/week)	2.48 ± 1.61	2.00 ± 1.41	0.5286 ^c^
Body mass (kg)	81.64 ± 8.51	63.97 ± 7.41	<0.0001 ^a^
Height (cm)	161.70 ± 5.21	161.10 ± 5.56	0.7893 ^a^
BMI (kg/m^2^)	31.14 ± 1.41	24.66 ± 2.81	<0.0001 ^a^
Lean body mass (kg)	43.02 ± 4.54	37.64 ± 3.53	0.0014 ^a^
Fat mass (kg)	36.37 ± 4.82	24.56 ± 5.31	<0.0001 ^a^
Android fat mass [%]	52.01 ± 2.63	44.71 ± 6.54	0.0001 ^b^
Gynoid fat mass [%]	50.35 ± 4.27	46.40 ± 3.76	0.0197 ^c^
COMP (ng/mL)	819.97 ± 267.62	735.06 ± 227.25	0.3845 ^a^
Adiponectin (µg/mL)	9.29 ± 3.57	11.15 ± 3.33	0.1862 ^a^
LMR	5.12 ± 1.89	4.70 ± 1.03	0.5663 ^b^
GLR	1.66 ± 0.51	1.89 ± 0.78	0.5995 ^c^

The differences between groups of women were investigated using ^a^ *t*-test, ^b^ Welch’s *t*-test, and ^c^ Mann–Whitney U test. BMI, body mass index; COMP, cartilage oligomeric matrix protein; LMR, lymphocyte-to-monocyte ratio; GLR, granulocyte-to-lymphocyte ratio.

**Table 2:** The results of the correlation analysis of COMP concentration with body composition and biochemical indices, with *p*-values adjusted using the Benjamini–Hochberg procedure (FDR).

Variable	Group with BMI Above Normal Range			Group with Normal BMI		
	Pearson’s Correlation Coefficient (r)	*p*-Value	Adjusted *p*-Values (FDR)	Pearson’s Correlation Coefficient (r)	*p*-Value	Adjusted *p*-Values (FDR)
Lean body mass (kg)	0.14	0.747	0.747	0.52	0.008	0.040
Fat mass (kg)	0.23	0.581	0.747	0.03	0.874	0.874
Adiponectin (µg/mL)	0.16	0.703	0.747	0.22	0.288	0.600
LMR	0.83	0.011	0.055	−0.19	0.360	0.600
GLR	−0.73	0.039	0.098	0.10	0.643	0.804

BMI, body mass index; LMR, lymphocyte-to-monocyte ratio; GLR, granulocyte-to-lymphocyte ratio.

## Data Availability

The datasets used and analyzed during the current study are available from the corresponding author upon reasonable request.
